# Custom-designed Small Animal focal iRradiation Jig (SARJ): design, manufacture and dosimetric evaluation

**DOI:** 10.1259/bjro.20190045

**Published:** 2020-03-06

**Authors:** Jothy Selvaraj, Graham Rhall, Mounir Ibrahim, Talat Mahmood, Nigel Freeman, Zennon Gromek, Grant Buchanan, Farhan Syed, Hany Elsaleh, Ben J. C. Quah

**Affiliations:** 1 Irradiation Immunity Interaction Lab, ACRF Department of Cancer Biology and Therapeutics, Canberra, ACT, Australia; 2 Medical Physics and Radiation Engineering, Canberra Health Services, Canberra, ACT, Australia; 3 The John Curtin School of Medical Research, Australian National University, Canberra, ACT, Australia; 4 Radiation Oncology Department, Canberra Hospital & Health Services, Canberra, ACT, Australia

## Abstract

**Objective:**

Preclinical animal models allow testing and refinement of novel therapeutic strategies. The most common preclinical animal irradiators are fixed source cabinet irradiators, which are vastly inferior to clinical linear accelerators capable of delivering highly conformal and precise treatments. The purpose of this study was to design, manufacture and test an irradiation jig (**s**mall **a**nimal focal i**r**radiation **j**ig, SARJ) that would enable focal irradiation of subcutaneous tumours in a standard fixed source cabinet irradiator.

**Methods and materials:**

A lead shielded SARJ was designed to rotate animal holders about the longitudinal axis and slide vertically from the base plate. Radiation dosimetry was undertaken using the built-in ion chamber and GAFChromic RTQA2 and EBT-XD films. Treatment effectiveness was determined by irradiating mice with subcutaneous melanoma lesions using a dose of 36 Gy in three fractions (12 Gy x 3) over three consecutive days.

**Results:**

The SARJ was tested for X-ray shielding effectiveness, verification of dose rate, total dose delivered to tumour and dose uniformity. Accurate and uniform delivery of X-ray dose was achieved. X-ray doses were limited to the tumour site when animal holders were rotated around their longitudinal axis to 15^o^ and 195^o^, allowing sequential dose delivery using parallel-opposed tangential beams. Irradiation of subcutaneous melanoma tumour established on the flanks of mice showed regression.

**Conclusion:**

SARJ enabled delivery of tangential parallel-opposed radiation beams to subcutaneous tumours in up to five mice simultaneously. SARJ allowed high throughput testing of clinically relevant dose delivery using a standard cabinet-style fixed source irradiator.

**Advances in knowledge:**

A custom designed jig has been manufactured to fit into conventional cabinet irradiators and is dosimetrically validated to deliver clinically relevant dose distributions to subcutaneous tumours in mice for preclinical studies.

## Introduction

Preclinical animal studies are crucial in the development of new therapeutic approaches. Animal models have been widely adopted for studying biological responses to various types of radiation modalities, doses and fractionation regimens, either alone or in combination with other treatments such as chemotherapy and immunotherapy. The importance of animal models in understanding the effects of radiation injury as well as in developing prophylactic and mitigatory measures against radiation exposure has been emphasized by the Centre for Medical Counter Measures for Radiation.^1^ For these studies, fixed-source orthovoltage cabinet irradiators with non-adjustable collimators are generally used^2,3^ that allow simultaneous irradiation of multiple specimens in their entirety due to a relatively large radiation beam size (around 40 cm in diameter). While these irradiators are ideal for some preclinical experimentations, they are not representative of radiation delivery approaches used in the clinic, especially when considering the precision and accuracy of targeting tumours and the ability to spare normal surrounding tissue(s) and critical organ(s) from receiving excessive radiation dose.^4–6^ Most modern linear accelerators that are employed for clinical use in general have a positional accuracy of ±2 mm and dose delivery uncertainty in the range of 1–3%.^4^ It is also worth noting that preclinical animal models, by virtue of their smaller size compared to humans, require even better positional precision and accuracy in the order of ±0.2 mm.^5,6^ Reproducing such a degree of high precision and accuracy in conventional cabinet irradiators is not only impractical but also next to impossible due to lack of on-board imaging systems and beam collimators.

Precise and accurate irradiation of the tumour while sparing the normal surrounding structures is of utmost importance when planning external beam radiation therapy. However, targeting and delivering a prescribed dose to a tumour established in a small animal model is not without its challenges. In general, a heterotopic tumour implanted on the hind leg of a small animal (usually a mouse) can be easily measured using callipers without the need for imaging and be adequately irradiated using a fixed-source cabinet irradiator. On the other hand, an orthotopic tumour -a preferred model for studying tumour invasion, metastatic potential and side-effects of therapies is difficult to measure and accurately irradiate without on-board image-guidance system, sophisticated treatment planning system and dosimetry evaluation tools. Furthermore, fixed-source cabinet irradiators deliver more or less the same dose to the tumour and the surrounding tissue(s), thereby making the contributions of mitotic cell death, immunogenic cell death, bystander effects and abscopal effects in tumour eradication^7–10^ difficult to distinguish from each other. Hence without precise, accurate and reproducible dosimetry, preclinical irradiation experiments involving small animals are often a waste of time, effort and cost.^11^

To overcome these challenges, small animal irradiators with on-board image-guidance systems and fixed and variable collimators have been developed.^12–15^ Some systems even offer bioluminescence imaging and treatment planning tools to accurately and precisely target and deliver the prescribed dose to the tumour while sparing the normal surrounding structures as much as possible. These systems, however, are prohibitively expensive and require specialised training to operate.

Realising the limitations of reproducing clinically relevant dosimetric patterns using a conventional fixed-source cabinet irradiator, we have designed a **s**mall **a**nimal focal i**r**radiation **j**ig (SARJ) for use in such irradiators. SARJ was designed to enable parallel-opposed tangential irradiation of subcutaneously implanted tumours on the flanks of mice while effectively sparing rest of their bodies. In this paper, we present the design, manufacture and dosimetric performance of SARJ. The shielding effectiveness of SARJ was validated using film dosimetry, the utility of which has been reported in several studies,^16–18^ and revealed advantages and limitations of the jig, which are discussed.

## Methods and materials

### SARJ design and manufacture

**Figure 1.**
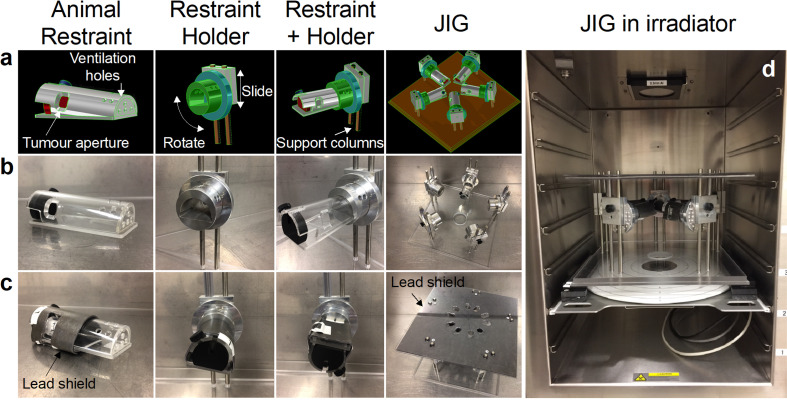
Design and manufacture of a Small Animal focal iRradiation Jig (SARJ). The SARJ was designed using SolidWorks software (1a). Mastercam software was used for dictating manufacture to an Okuma mill, Mori Seiki lathe/mill and Sodick wire cutter electronic discharge machine for the making of the various components (1b). Animal restraints were shielded with 2.1 mm lead to refine tumour targeting and the whole jig was shielded with further with 3 mm lead with oval holes aligned above each targeted tumour site (1c). The jig was designed and manufactured to fit in a standard-sized cabinet irradiator (1d).

The jig was designed using SolidWorks (SolidWorks Corporation, MA) computer-aided design software (Figure 1a). Mastercam (CNC Software Inc., MA, USA) software was used for computer-aided manufacturing that was implemented with an Okuma mill (Okuma, Oguchi, Japan), a Mori Seiki lathe/mill (DMG Mori Seiki, Nagoya, Japan) and a Sodick wire cutter electrical discharge machine (Sodick, Yokohama, Japan). The mouse restraints, having 4.5 mm ventilation holes at its anterior end and a 10 mm diameter aperture posterolaterally to accommodate a single tumour established in the right hind flank of the mice, were manufactured from 2.5 mm thick acrylic (Figure 1a, b). Holders for each restraint were designed to rotate at 15° increments about the longitudinal axis and to slide vertically on two 7 mm diameter support columns (Figure 1a). The holders and columns were manufactured from aluminium (Figure 1b). The holder design enabled 180° rotation of the animal allowing delivery of parallel-opposed radiation beams and their vertical movement allowed dose rate variation taking advantage of the inverse-square law effect. The latter in turn enabled delivery of different doses to the tumour for the same irradiation time within the limitations of beam divergence and SARJ shielding (mentioned below). The entire assembly was comprised of five restraints with holders placed in a circular arrangement and was attached via the vertical support columns to a 15 mm thick acrylic base plate (Figure 1a and b last panels). The mouse restraints were shielded with 2.1 mm of lead with a hole cut out around where the tumour was to be positioned (Figure 1c, first three panels). This shielding corresponded to three half-value-layers (HVLs) for 225 kV X-ray attenuating 87.5% of the radiation beam intensity, or in other words a leakage of 12.5% of the incident beam intensity on the shield. An additional 3.0 mm lead plate with oval holes aligned above the tumour positions of each restraint formed the roof of the jig (Figure 1c, last panel), effectively providing seven HVLs of beam attenuation outside the target area, bringing down the radiation leakage to 0.8%. When fully assembled, the dimensions of SARJ were 34.5 cm (depth) x 34.5 (width) x 22.5 cm (height) allowing it to fit into standard cabinet-style irradiators such as the RS 2000 (Radsource, Buford, GA) and the MultiRad225 (Faxitron Biotics, Tucson, AZ) (Figure 1d).

### SARJ radiation dosimetry

**Figure 2.**
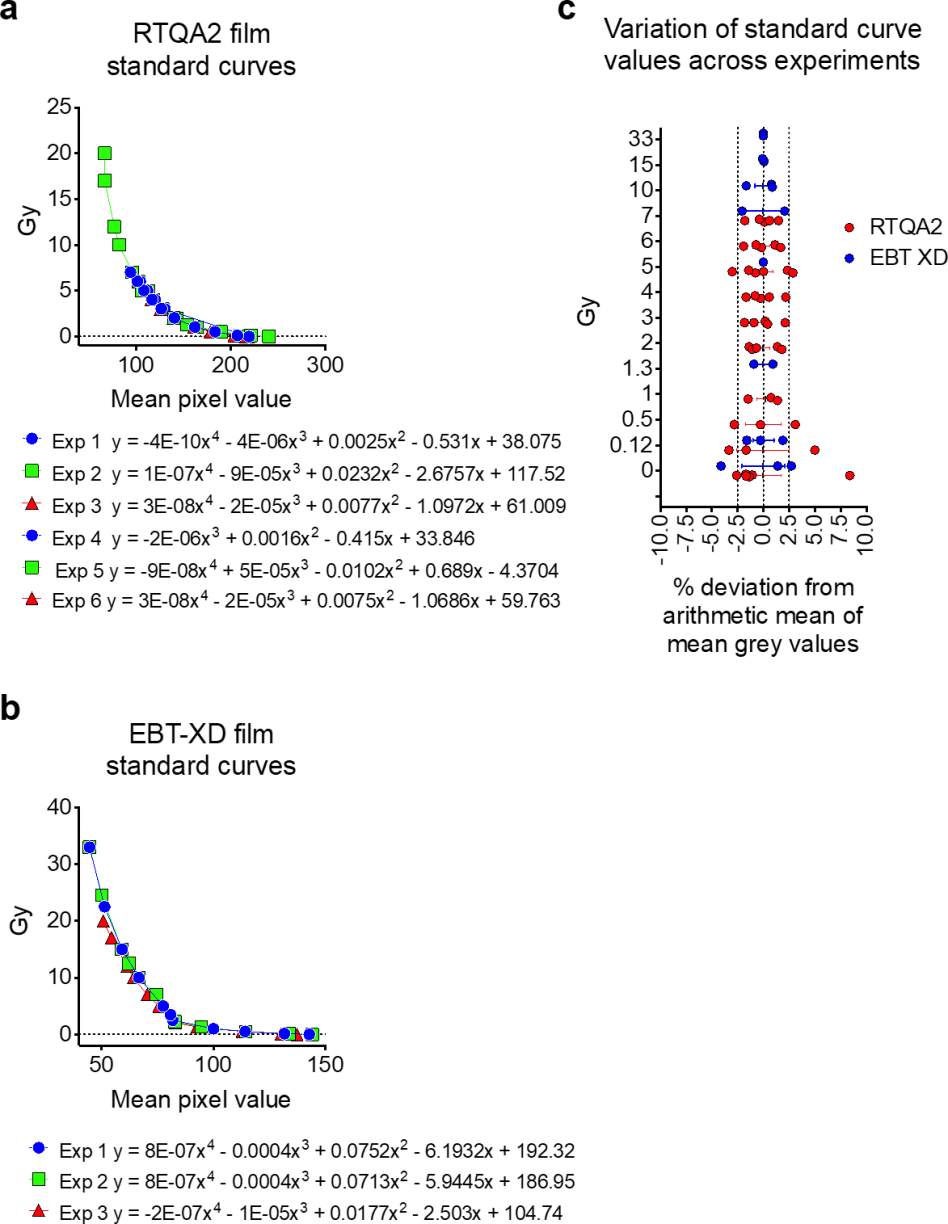
HD standard curves and dose measurementcomparisons using RTQA2 and EBT-XD Gafchromic film. Standard curves from nine experiments using either RTQA2 (*n* = 6) (**a**) or EBT-XD (*n* = 3) Gafchromic film (**b**), generated by exposing different pieces of film to discrete X-ray doses ranging from 0 to up to 33 Gy (assessed using the built-in MultiRad225 dosemeter). Plots show X-ray dose against mean pixel values of red channel data of the film as described in the methods. Polynomial equations of either third or fourth ordered were fitted to each curve and are listed for each experiment. (**c**) Deviation from the mean of mean pixel values at each X-ray dose across nine standard curves were calculated to evaluate inter experimental variability (results are shown for doses with *n* > 2 only).

A MultiRad225 X-ray irradiator, with a beam coverage range of 9–40 cm in diameter and an adjustable sample to source distance of 13–65 cm (Faxitron Biotics, Tucson, AZ) operating at 225 kV and 17 mA with a 0.5 mm Cu filter in addition to 2.0 mm of inherent Beryllium filter, corresponding to 1.3 mm of HVL in copper, was used for SARJ dosimetry tests. The distance of the radiation source to the anticipated centre of the tumour was standardised at 37 cm. Shielding adequacy and tumour dose uniformity was validated with either the built-in parallel-plate ion chamber and/or Gafchromic films which have a near-tissue equivalence for dosimetric purposes and have been used previously in a number of similar dosimetric studies, validating this approach.^19–23^ The built-in dosemeter was validated without the SARJ in the cabinet by an independent dosimetry system using the AAPM TG61 protocol with a 0.6 cc Farmer ion chamber without the build-up cap.^24^ The 0.6 cc Farmer ion chamber was cross-calibrated against our reference ion chamber calibrated by an accredited dosimetry calibration laboratory. Gafchromic RTQA2 films, with a dynamic dose range of 0.02–8.0 Gy (Ashland Advanced Materials, Bridgewater, NJ), were chosen when qualitative and simultaneous quantitative analysis were required; whereas Gafchromic EBT-XD films, with a dynamic dose range of 0.1–60 Gy (Ashland Advanced Materials, Bridgewater, NJ), were used when mainly quantitative analysis was required and particularly in the wax phantoms (see below). For quantitative radiation dose analyses, Gafchromic film was used similar to methods previously described.^25^ Briefly, exposed GAFChromic film strips from either in-air measurements, phantom measurements or standard curve measurements (described in separate sections below) were scanned 24 h after exposure in RGB format at 48-bit and at 600 dpi resolution using a Canon CanoScan 9000FMarkII flatbed scanner (Canon, Japan). ImageJ software was used to split the RGB image into individual channels and the red channel data were used for analysis. The mean pixel values at the centre of the exposed region of the films (using a 50 × 50 pixel square) were then used for analysis. To calculate dose in Gy from mean pixel values, polynomial interpolation from Hunter-Driffield (HD) standard curves was used. HD standard curves were generated by exposing different pieces of film to discrete X-ray doses and included 0, 0.1, 0.5, 1, 2.5, 3, 3.5, 4, 5, 6, 7, 10, 12, 15, 17, 20, 22, 24, 30 and 33 Gy, assessed using the built-in MultiRad225 dosemeter. HD curves were generated for each experiment using film from the same film sheet as that used for in-air and phantom dose calculations to account for film batch and inter experimental variability (note, not all dose ranges listed above were used for all experimental data curves, but rather the dose range was tailored for each experiment to cover the experimental range). GraphPad Prism 8.0 software was used to plot the mean pixel value of film against radiation dose, and a third- or fourth-order polynomial equation was fitted to the data to allow polynomial interpolation of film dose exposure using mean pixel values. Figure 2a and b shows the standard curves of the different experiments using both types of film. Also, included in this figure are the polynomial equations used to calibrate the film doses for each experiment. It was found the deviation of HD curve data points across experiments at the X-ray doses used was typically within ±2.5% of the mean (Figure 2c).

### SARJ in-air radiation dosimetry

**Figure 3.**
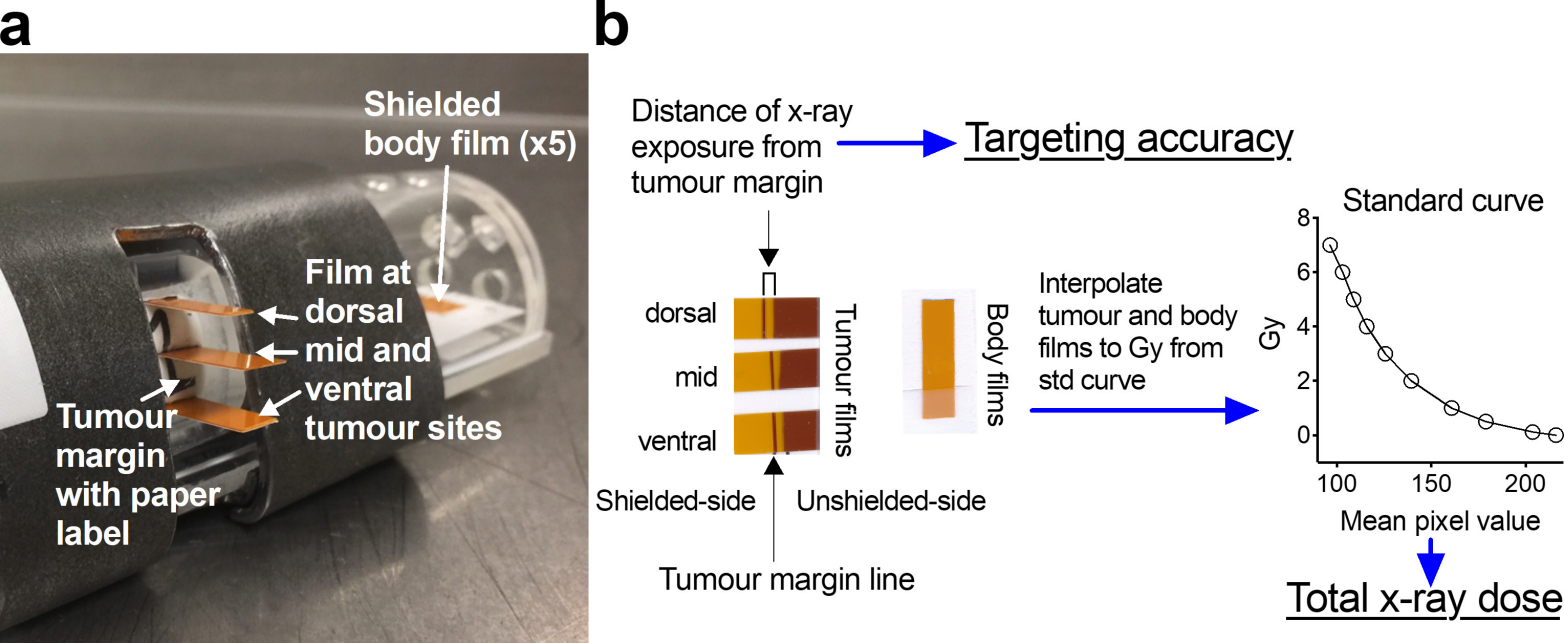
Gafchromic film placement and use for assessing X-ray targeting accuracy and total dose. Rectangular strips of RTQA2 Gafchromic film were placed at the dorsal, midline (mid) and ventral positions of the tumour site through a paper label placed to conform with the inner boundaries of the mouse restraint (defined as the tumour margin and marked with a pen line on the film) (**a**). Film was also placed along the cranio-caudal axis of the mouse restraint that was within the shielded area (Shielded body film) (**a**). Exposed film was used to assess X-ray targeting accuracy (by measuring distance from tumour margin) and X-ray total dose (by polynomial interpolation from a dose *vs* mean pixel value standard curve as described in the methods) (**b**).

For in-air dosimetric evaluation along the tumour, small rectangular strips of Gafchromic film were placed perpendicularly along the vertical axis of the anticipated tumour site at three positions—dorsal, midline and ventral—with films facing the radiation source (Figure 3a). The tumour margin was defined as the surface conforming to the inner boundaries of the mouse restraint at the tumour site aperture (Figure 3a). The films, attached through a paper label, were oriented spanning both inwards and outwards from the tumour margin (marked with a pen line on the film) to assess whether the lead shielding adequately exposed the tumour area while preventing radiation exposure to the rest of the body (Figure 3a and b). To assess X-ray targeting accuracy, the distance of exposed film from the tumour margin was calculated using digital callipers (Figure 3b). The films were also assessed for total X-ray dose using polynomial interpolation as described above (Figure 3b).

**Figure 4.**
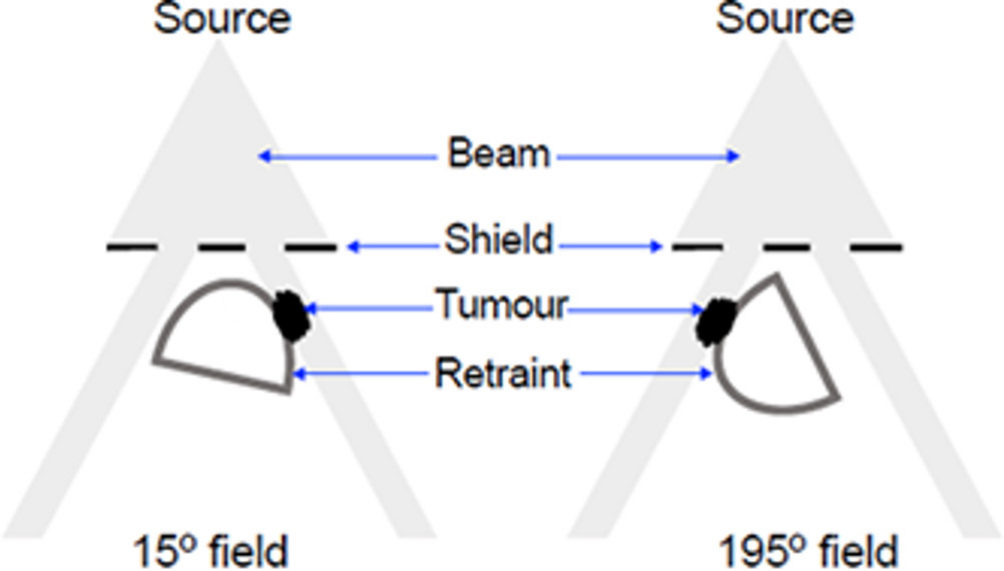
Schematic diagram showing tangential irradiation of hind flank tumours at 15 and 195° as seen from the centre of the jig. Rotation of the SARJ animal holders allowed placement of the right hind flank tumour region in the 15^o^ field or the 195^o^ field. Lead shielding above and around (not shown) the animal restraint limited dose to the tumour region.

All analyses were performed for both anterior and posterior (*i.e.,* right medial and right lateral tangents relative to the anticipated animal geometry) beams separately by rotating the animal restraint to 15° and 195° from the horizontal axis as shown schematically in Figure 4. To measure out-of-field doses, Gafchromic film strips were also placed outside the target area along the cranio-caudal axis of the mouse restraint (*i.e.,* shielded body film, Figure 3a and b). Each measurement was repeated several times (*n* > 3) to obtain mean dose values at each position. Statistical analysis was performed using GraphPad Prism 8.0 (GraphPad Software, Inc, San Diego, CA).

### SARJ wax phantom radiation dosimetry

**Figure 5.**
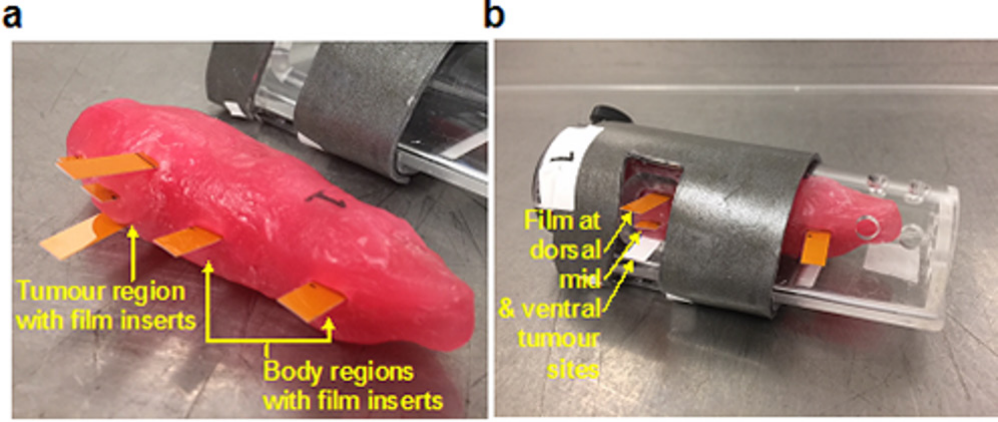
SARJ wax phantoms with Gafchromicfilm placement for x-ray dosimetry. Metrowax phantoms were made for each animal restraint resembling the approximate size of a mouse and included a protruding “tumour” in the right hind flank (a). Rectangular strips of Gafchromic film were placed at different body regions including dorsal, midline (mid) and ventral positions of the tumour site and shielded body regions (a, b). The wax phantoms were designed to fit in the animal restraints as a live mouse would (b).

Mouse phantoms, with a protruding right hind flank tumour (with an approximate dimension of 7 x 7 x 7 mm) were prepared from dental modelling wax composed of paraffin and microcrystalline waxes (Metrowax, Metrodent Limited, United Kingdom) (Figure 5a). The dimensions of the phantoms were ~85 mm in length and ~22 mm in diameter. The density of the Metrowax was reported to be 0.85 g/cc.^26^ Metrowax has been shown to be tissue equivalent using Monte Carlo simulations.^27^ Small apertures throughout the phantom were positioned to allow insertion of Gafchromic films (Figure 5a and b) to monitor internal dose using interpolation from a HD curve, as described above. The tumour margin in the phantom was defined as the region of confluence of the wax tumour protrusion and the main body of the phantom. This region coincided with the inner boundaries of the animal restraint aperture during testing (Figure 5b).

### SARJ-mediated melanoma treatment

B16-F10^OVA^ cells (5 × 10^5^), suspended in 30 µL of saline, were injected subcutaneously in the right hind flank of 12 C57BL/6J mice (provided by the Australian Phenomics Facility, ANU) and were left for 8 days to establish into 8 x 8 mm tumours. Mice were then anaesthetised by intraperitoneal injection of ketamine 100 mg ml^−1^ and xylazine 10 mg ml^−1^. Six anaesthetised mice were placed in the SARJ, and their tumours were irradiated tangentially using parallel opposed beams to a dose of 12 Gy (6 Gy through each beam) prescribed at the centre of the tumour. Radiation dose was repeated on days 9 and 10 post-tumour cell injection, whereas six control mice received no irradiation. Mice were subsequently monitored at 2 days intervals and tumour width (W) and length (L) was measured using digital callipers. The tumour volume was then calculated using the formula (W^2^ x L)/2. Mouse well-being was monitored daily and tumour scores were assessed using a tumour study scoring and well-being index (Supplementary Material 1) over the course of the experiment. Mice identified to suffer unduly based on these scores were euthanised immediately by cervical dislocation to ensure humane end points. The study was complete within 35 days at which point remaining mice were euthanised (no animals were found dead during this study). Statistical analysis on tumour growth was performed using GraphPad Prism 8.0 (GraphPad Software, Inc, San Diego, CA). Mice were housed and handled according to the guidelines of the ANU Animal Experimentation Ethics Committee. This included them being maintained in sterilised isolated ventilated cages containing corn cob bedding, cardboard cylinder enrichment, and sterile food and water. All animal handling staff underwent and met the requirements of ANU-specific animal handling training.

## Results

### SARJ design and manufacturing

Many preclinical animal models use protruding tumours established subcutaneously in the hind flank.^28^ These tumours are amenable to treatment with tangential beams using appropriate shielding to restrict dose to lesions protruding from the body surface. We, therefore, set out to design a mouse platform or jig using enclosed animal restraints with a 10 mm aperture corresponding to the right hind flank to expose the protruding subcutaneously established tumours (Figure 1). The animal restraint was designed to fit into a rotating holder and, therefore, allowed parallel-opposed tangential radiation beam delivery at 15° and 195° from the horizontal axis (Figure 1). In addition, SARJ was designed to fit five animal restraints and holders aligned symmetrically in a radial arrangement to allow simultaneous and uniform radiation dose delivery to the individual tumours (Figure 1). Lead shielding was placed around the tumour aperture of each animal restraint (Figure 1), whereas an additional umbrella lead plate, with holes placed for both 15° and 195° beams, was placed on top of the holders to further limit radiation exposure to the animals (Figure 1). The final assembly was able to fit into a standard cabinet-style irradiator (Figure 1d).

### SARJ in-air radiation dosimetry

**Figure 6.**
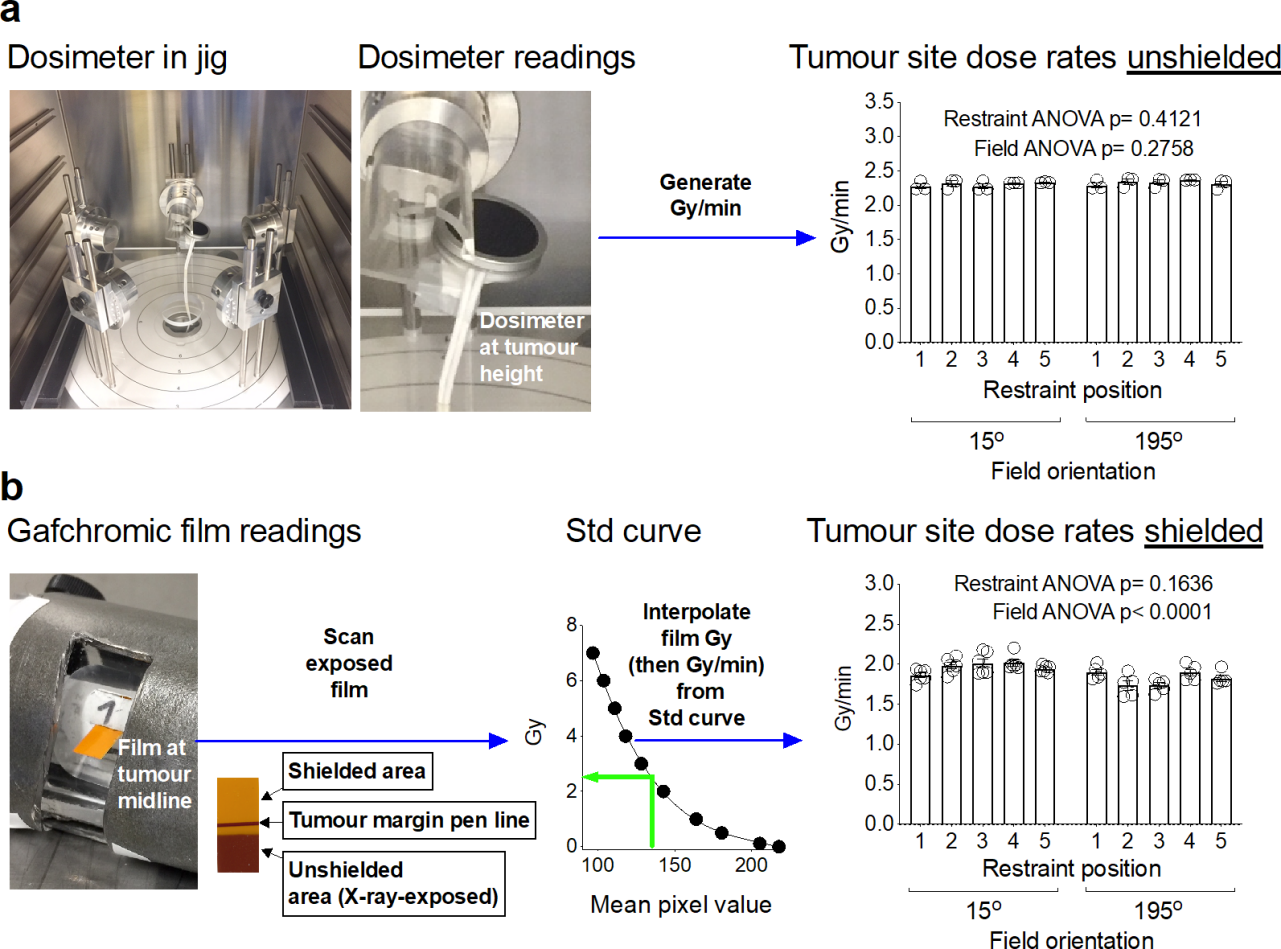
SARJ tumour site dose rates. The built-in irradiator dosemeter was used for assessing initial dose rates at each tumour positions for both the 15° and 195° beams (**a**). RTQA2 Gafchromic was used to assess radiation dose delivery at the midline of tumour position (**b**). Film dose was determined by polynomial interpolation via a HD standard curve (as described in the materials and methods) and dose was converted to dose rate by dividing dose with beam-on time. Data was from *n* > 3 and shows mean values and standard error of mean. Statistical analysis was performed using 2-way ANOVA.

Initial dosimetric analysis to assess uniformity of dose rates was carried out at each of the five tumour sites for each of the beams (15° and 195°) in the unshielded jig. The built-in dosemeter of the cabinet irradiator was used for dose rate measurements (Figure 6a) and revealed statistically equivalent dose rates at each tumour site and for both the beams (Figure 6a). As the built-in dosemeter could not fit in the fully assembled jig, Gafchromic RTQA2 film was used to measure the dose using polynomial interpolation from a film standard curve (as described in the methods) and converted to dose rate, in the shielded jig (Figure 6b). Analysis of the film after X-ray exposure demonstrated statistically equivalent dose rates at each tumour position for each beam. There was, however, a statistically significant difference in the dose rate for individual beams, with the 195° beam having a (7%) lower average dose rate compared to the 15° beam, possibly due to the geometric changes for each field in the shielded jig.

### SARJ tumour targeting and effectiveness

**Figure 7.**
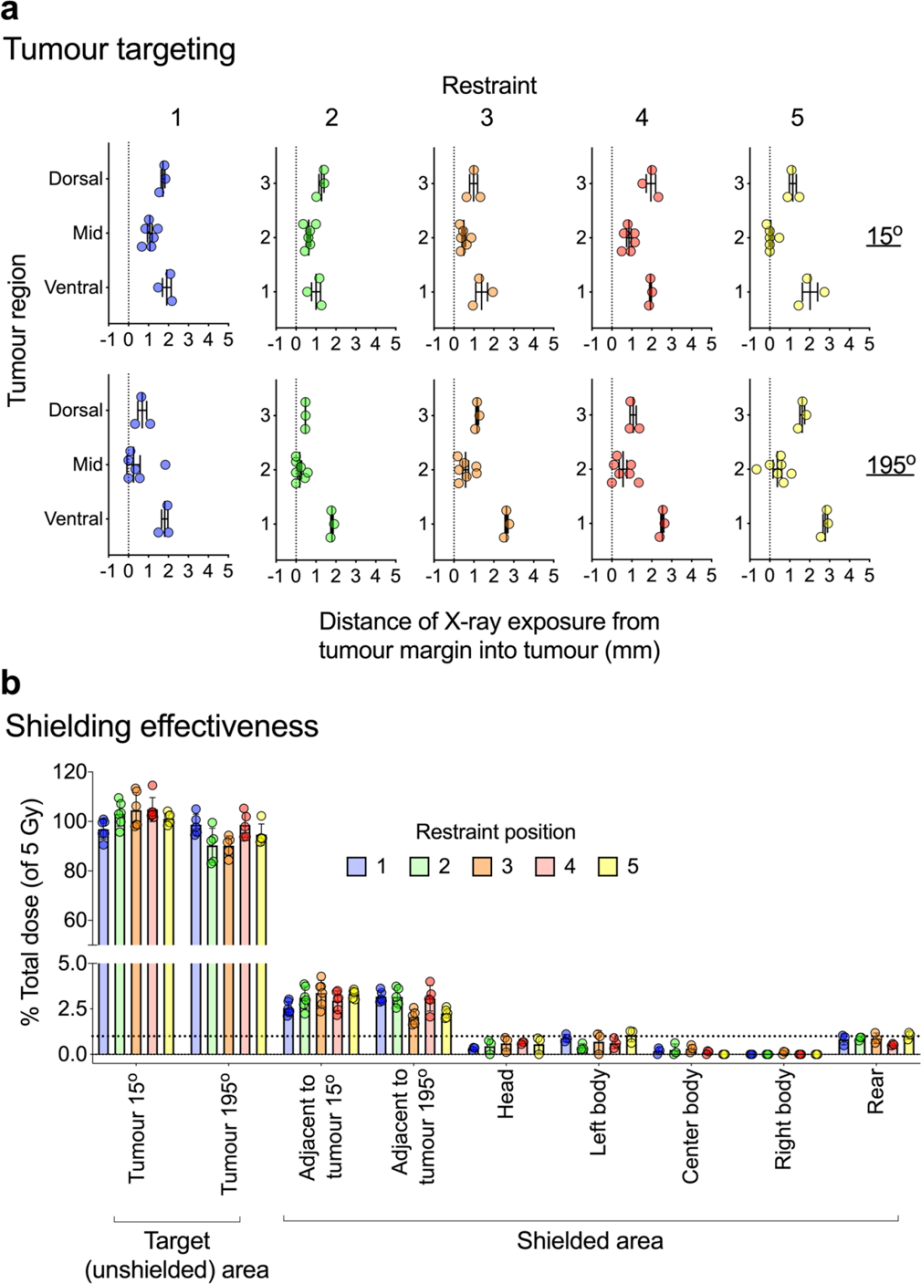
SARJ tumour targeting and shielding effectiveness. X-ray irradiation was performed as in Figure 6. X-ray beam accuracy at the margin of the tumour site was assessed with RTQA2 Gafchromic film placed perpendicular to the anticipated body surface at the dorsal, midline and ventral tumour sites (Figure 3). The tumour margin was defined as the surface conforming to the inner boundaries of the animal restraint and this is where a paper label was attached to position the pieces of film (Figure 3a). Film was placed through the paper label spanning both inwards and outwards from the tumour margin (which was marked with a pen line for reference) (Figure 3a and b). After X-ray exposure, the distance from the tumour margin (pen line) to beam-exposed film was assessed with digital callipers (Figure 3b) and plotted at each tumour position and after exposure from each beam (15° and 195°) (Figure 6a). X-ray dose was also assessed from the RTQA2 Gafchromic film placed at midline of the tumour site as well as from film placed at the head, body and rear of the shielded animal restraint (calculated as in Figure 6b) and is displayed as % dose of the total 5 Gy prescribed at the tumour site (b). Dose at the unshielded tumour site and adjacent shielded region were calculated 2–3 mm externally or internally from the tumour margin marked on the film respectively. Data are calculated from *n* > 3 and shows mean values and standard error of mean. Dotted line in (b) is 1% Total dose.

The distinct colour change of the exposed Gafchromic RTQA2 film enabled its use in assessing the accuracy of radiation beam delivery at the tumour sites relative to the adjacent shielded areas. The distance from the tumour margin to the region of film exposure was measured to evaluate the adequacy of lead shielding (Figure 3). With Gafchromic film placed at the dorsal, midline and ventral sites of the tumour region, we could assess radiation exposure across these sites. Since these films were placed along the curved aperture of the tumour margin (which we have defined as the surface conforming to the inner boundaries of the animal restraint and is anticipated to be where the actual tumour would meet normal tissue), we aimed to target the beam closest to this margin at the midline of the tumour. Indeed, compared to the dorsal and ventral tumour areas, the midline of the tumour site received radiation exposure closest to the margin (Figure 7a). Radiation exposure at the tumour midline was typically within 0–1 mm from the tumour margin with little penetration into the adjacent body areas beyond the margin (Figure 7a). These findings were consistent across all the tumour sites at each restraint for the individual beams (15° and 195°).

To further calculate the actual dose penetrating the shielded area adjacent to the tumour site, the tumour midline film was assessed for total dose and presented as a percentage of the anticipated total dose at the tumour midline (Figure 7b). Furthermore, films were also placed within the shielded area corresponding to the locations of the head, body and rear of the animal to assess the percentage of dose received in these areas (Figure 7b). While the unshielded tumour area received ~100% of the total anticipated dose, the shielded areas adjacent to the tumour margin received less than 5% of the total dose, whereas areas corresponding to the head, body and rear of the animal received less than 1.3% of the total dose.

### SARJ wax phantom radiation dosimetry

**Figure 8.**
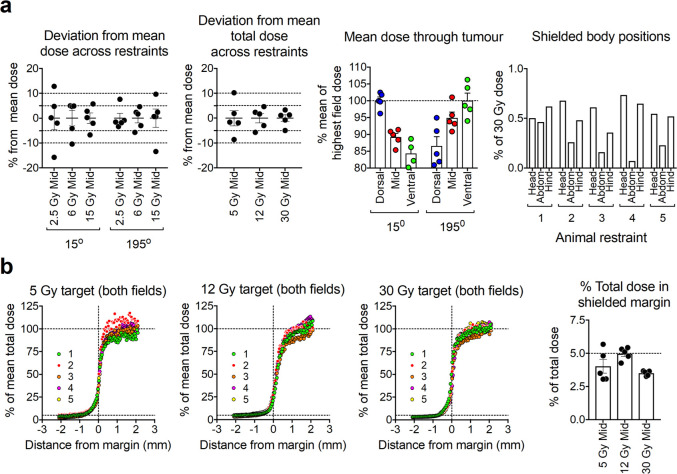
SARJ Dosimetry in wax phantoms. SARJ, **s**mall **a**nimal focal i**r**radiation **j**ig. Gafchromic film was used to monitor internal X-ray dosage in wax phantoms Figure 5 using interpolation from a HD standard curve as described in the materials and methods (a). Uniformity of dose within the midline of the wax tumour across restraints was assessed at prescribed X-ray dosages of 2.5, 6 and 15 Gy at each field (15° or 195°) (Figure 8a first panel) or after combining the total dose delivered from both fields together (15° and 195°) (Figure 8a second panel). Tumour depth-dependence of the dose was measured with film placed at dorsal, mid and ventral sites though the phantom tumour at each field (Figure 8a third panel). Percentage tumour dose from both fields at three shielded body positions (head, body and rear) was also assessed after a prescribed dose of 30 Gy to the tumour midline (a third panel). Accuracy of X-ray dose to tumour was assessed by plotting the distribution of dose across the tumour margin (defined as where the wax tumour meets the main body of the wax phantom, which was marked with a pen line on the film for reference) after prescribed doses of 5, 12 and 30 Gy (with half of each dose being delivered through each beam) (Figure 8b first three panels). Penetration of X-rays 1 mm from the tumour margin within the shielded side-was assessed by calculating the percentage total dose after prescribed doses of 5, 12 and 30 Gy (with half of each dose being delivered through each beam) (b last panel). Error bars are standard error of mean.

Compared to in-air dosimetry, the presence of biological tissue(s) within the path of radiation beam in a standard cabinet-style irradiator without any capability of beam collimation may generate additional scatter radiation and may alter radiation dose deposition. Therefore, to further assess SARJ dosimetric performance, mouse phantoms made of Metrowax which has near-biological tissue equivalent radiation beam attenuation properties^27^—with a protruding tumour in the right hind flank were manufactured (Figure 5). Rectangular strips of Gafchromic film were inserted in the tumour and parts of the body to measure the internal dose (Figure 5). Gafchromic film was used for dose calculations and the results were interpolated using a polynomial equation fitted to HD standard curves, as described in the material and methods section (Figure 8a). The mouse phantom was irradiated to doses of 5, 12 and 30 Gy, split into parallel-opposed tangential beams (15° and 195°), prescribed to the centre of the wax tumour. Tests performed to determine radiation dose deviation at the tumour midline to estimate tumour dose uniformity at each restraint for the individual beams (Figure 8a, first panel) demonstrated typical dose delivery within 10% of the mean dose across the range of delivered dose (Figure 8a, first panel). Calculating the total delivered dose, by adding the contributions from both the tangential beams, resulted in decreased dose variability to the tumour midline across the restraints to typically within 5% of the mean dose (Figure 8a, second panel). Overall, standard error of mean total dose (from both 15° and 195° beams) across the restraints was 4.6%. To assess the tumour depth-dependence of the dose, measurements were collected parallel to the field at the dorsal, midline and ventral sites though the phantom tumour using film inserts at these sites (Figures 3 and 8a third panel). This revealed a 5–10% drop in dose from the X-ray tumour entry site to the tumour midline and a further 5–10% drop from tumour midline to the tumour exit site (Figure 8a third panel). This trend occurred with both fields across all the five phantom tumour sites in each animal holder (Figure 8a, third panel). In addition, radiation dose to the head, abdomen and rear of the mouse phantom, assessed by delivering 15 Gy through each tangential beam (30 Gy total) to the tumour midline, was calculated to be less than 1% (mean 0.46%) of the dose prescribed to the tumour region (Figure 8a last panel).

To further explore the accuracy of SARJ in targeting the tumour region of the mouse phantom, dose distribution across the tumour margin was assessed at three doses employing both the tangential beams (Figure 8b). This analysis demonstrated that the radiation dose dropped sharply at the tumour margin, typically being ≤5% of the total dose detected 1 mm into the shielded area from the margin (Figure 8b). The full dose was delivered within 1–2 mm from the unshielded margin to the tumour (Figure 8b) with the beam penumbral width, measured between 80 and 20% of the cross-profile, being around 2 mm (Figure 8b first three panels).

Overall, SARJ accurately delivered the prescribed dose to the tumour region of all five tumour positions with ≤5% uncertainty and around 0.5% of the prescribed dose was delivered to the adjacent and remote shielded areas of the body. The main limitation of SARJ appeared to be variability in dose delivery during simultaneous exposure to the five individual tumour sites, and thus comparing radiation doses within 10% increments would not be advisable.

### SARJ-mediated melanoma treatment

**Figure 9.**
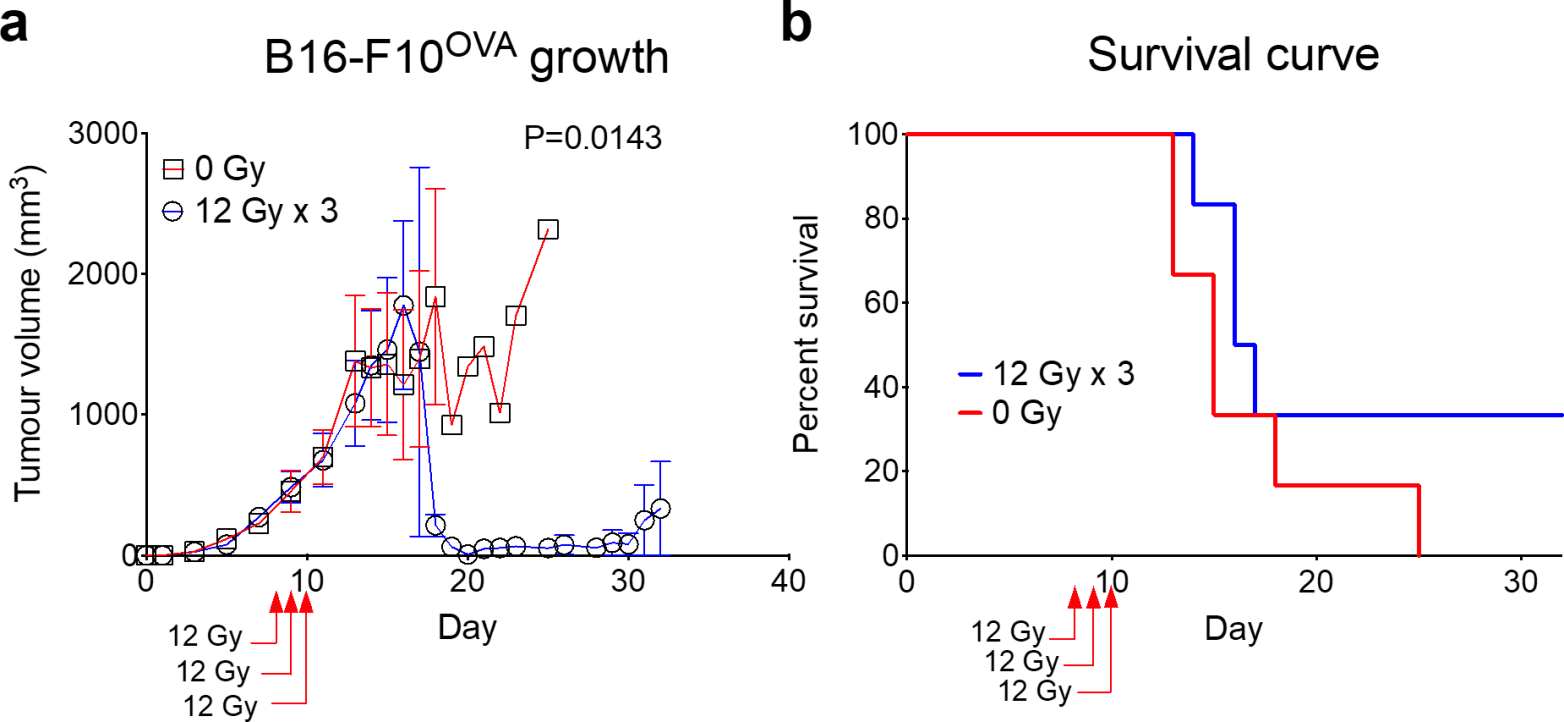
Treatment of advanced melanoma using the SARJ. SARJ, **s**mall **a**nimal focal i**r**radiation **j**ig. Day-8 B16-F10^OVA^ tumours were established s.c. in the right hind flank of C57BL/6J mice. Mice were anaesthetised and placed in the SARJ and irradiated tangentially with 6 Gy through each parallel opposed beam to give a total dose of 12 Gy. Irradiation treatment was repeated for another 2 consecutive days to give a total of 12 Gy x 3. Control mice received no irradiation. Mice were monitored for tumour growth over time (a). Survival of mice over time was also determined (b). Error bars are standard error of mean with *n* = 6, and statistical analysis was performed using the Mann–Whitney test.

Following mouse wax phantom dosimetric analysis, the SARJ was tested by focally irradiating advanced B16-F10^OVA^ melanoma tumours measuring about 8 mm in diameter that were established on the right hind flanks of C57BL/6J mice. These tumours were typically found to have a progressive and locally destructive growth pattern in the control mice. Tumours were irradiated with tangential parallel-opposed beams to a dose of 12 Gy per day over 3 consecutive days, with each beam delivering 50% of the prescribed dose. The tumour size of the surviving mice was then measured over time and mouse survival was recorded (Figure 9). A significant reduction in tumour burden was observed in surviving mice irradiated in the SARJ compared to the control mice receiving no treatment (Figure 9a). The treatment response lasted for about 10 days after which tumour recurrence was observed. Corresponding to tumour burden reduction, a survival benefit was observed in treated mice compared to the controls (Figure 9b).

## Discussion

The purpose of this study was to design, manufacture and perform dosimetric analysis of a SARJ that can precisely and accurately irradiate tumours established in the hind flank of mice using a standard cabinet-style X-ray irradiator. The manufactured SARJ was able to fit within a MultiRad225 cabinet irradiator and allowed simultaneous tangential irradiation of up to five tumour-bearing mice. When measured with an ion chamber, there was no statistically significant difference in radiation dose delivered at the tumour region of the different mouse restraints for both the tangential beams in unshielded conditions. However, X-ray film measurements in the lead-shielded jig revealed statistically significant difference in radiation dose delivery between the two parallel opposed tangential beams and were potentially attributed to the geometrical changes introduced due to the rotation of the restraints, film placement in the animal holders and shield contributions. For example, the film placement at the tumour site did not have to be changed at each field measurement, unlike the chamber measurements (performed in the unshielded jig) which required reorientation of the chamber at each field. Furthermore, since the film measurements were obtained in the shielded jig (which was not possible with the chamber), the shielding at the different field orientations may have contributed to the dose rate changes at each field. Since the film measurements represent a more accurate representation of the tumour site orientation and shielding arrangements during tumour treatments, we have relied on these to estimate dose. In general, 195° beam delivered a slightly lesser dose compared to the 15° beam. This can be compensated for by increasing the beam-on-time during dose delivery through 195° beam to match the dose of the opposing beam. The dose measurements at 2.5, 6 and 15 Gy with tangential beams showed variation within ±10% across the tumour sites, suggesting a limitation of the jig when comparing the impact of dose differences within this variation. Given, the generally observed sigmoidal relationship between radiation dose and tumour response,^29^ this variation might introduce considerable uncertainty in the experimental results. In contrast, cutting-edge small animal image-guided irradiators with a dedicated TPS with Montecarlo algorithms give dose reproducibility of ≤5%^30^ with delivery accuracy of ±0.1 mm.^12^

In-air out-of-field radiation dose delivered adjacent to the tumour region was less than 5% of the in-field dose, whereas elsewhere over the entire extent of the mouse holder it was less than 2% of the in-field dose. The higher out-of-field dose adjacent to the tumour site was presumably due to the increased radiation scatter and penumbral contribution of the beams. Dose measured at the head, abdomen and rear of the wax phantom when delivering 30 Gy was around 0.15 Gy (0.05%) with shielding. This is comparable to dose distributions observed in clinical practice when using highly conformal radiotherapy techniques, such as intensity modulated radiotherapy (IMRT) and volumetric modulated arc radiotherapy (VMAT). The ability of SARJ to result in clinically relevant radiation dose distributions would be essential for studying abscopal effects of radiotherapy.

The distance between the beam edge and the tumour margin was found to be between 1 and 2 mm for both the tangential beams. The beam penumbral width measured between 80 and 20% of the cross-profile was around 2 mm. These parameters were found to be reasonably adequate for irradiation of subcutaneous tumours established in the flank through tangential beams as demonstrated by clear regression of some of the advanced B16 melanoma flank tumours.

Despite potential utility of SARJ in preclinical studies, without on-board imaging systems and three-dimensional (3D) dose calculation and evaluation tools, it is impractical and impossible to estimate the accuracy of dose delivery generated within SARJ in 3D. While we have used radiochromic films placed around the tumour site to validate dose accuracy and delivery (data not shown), such measurements are not a substitute for image-guided radiation delivery, dose calculation and evaluation tools. SARJ, by virtue of its design, is impractical for irradiating orthotopic tumour models other than skin cancer models where tumour deposits are established subcutaneously in the right hind flank, as many clinically relevant deeply embedded orthotopic tumour models would require 3D image-based treatment planning and delivery to effectively spare the surrounding normal tissue(s). Despite this, SARJ can be used for any type of tumour established in the right hind flank of mice and is currently being used for colorectal cancers, breast cancers and melanomas models. While we limited this study to irradiation of tumours up to 10 mm in diameter, larger tumours could be irradiated if required by modifying the animal restraint and its shielding and be used in the current holder infrastructure.

## Conclusion

Employing parallel-opposed tangential beams, clinically relevant radiation dose distributions were achieved using SARJ for up to five specimens simultaneously. SARJ was accommodated into a cabinet-style irradiator without requiring any modifications to the interior of the irradiation unit. It also provided effective shielding to the normal tissue outside the tumour. While SARJ is not a replacement for expensive image-guided small animal irradiators capable of delivering precise and highly conformal treatments, it was found to be a cost-effective and practical add-on to a standard cabinet-style irradiator.
